# Tissue Interlocking Dissolving Microneedles for Accurate and Efficient Transdermal Delivery of Biomolecules

**DOI:** 10.1038/s41598-019-44418-6

**Published:** 2019-05-27

**Authors:** Shayan Fakhraei Lahiji, Youseong Kim, Geonwoo Kang, Suyong Kim, Seunghee Lee, Hyungil Jung

**Affiliations:** 1 Department of Biotechnology, Building 123, Yonsei University, 50 Yonsei-ro, Seodaemun-gu, Seoul, 03722 Republic of Korea; 2 Juvic Inc., 272 Digital-ro, Guro-gu, Seoul, 08389 Republic of Korea

**Keywords:** Biotechnology, Health care

## Abstract

The interest in safe and efficient transdermal drug delivery systems has been increasing in recent decades. In light of that, polymeric dissolving microneedles (DMNs) were developed as an ideal platform capable of delivering micro- and macro-biomolecules across the skin in a minimally invasive manner. A vast majority of studies, however, suggest that the shape of DMNs, as well as the elastic properties of skin, affects the delivery efficiency of materials encapsulated within DMNs. Likewise, in dynamic tissues, DMNs would easily distend from the skin, leading to inefficient delivery of encapsulated agents. Thus, herein, to improve delivery efficiency of DMN encapsulated agents, a novel hyaluronic acid backbone-based tissue interlocking DMN (TI-DMN) is developed. TI-DMN is simple to fabricate and significantly improves the transdermal delivery efficiency of encapsulated materials compared with traditional DMNs. The enhanced tissue interlocking feature of TI-DMN is achieved through its sharp tip, wide body, and narrow neck geometry. This paper demonstrates that TI-DMN would serve as an attractive transdermal delivery platform to enhance penetration and delivery efficiency of a wide range of biomolecules into the body.

## Introduction

Skin, the largest organ of the human body, is an attractive target for transdermal delivery of micro- and macro-biomolecules^1–3^. Stratum corneum, however, made of dozens of dead cell layers^4^, significantly reduces the penetration and delivery efficiency of macromolecules across the skin^5–8^. As a result, in most cases, a higher dosage is applied, which increases the formulation costs and may lead to unpredictable side effects^9^. Another convenient route, the oral administration, on the other hand, is affected by the first pass metabolism of gastrointestinal tract^10^, reducing the delivery efficiency of most biomaterials before reaching the target sites^5^. Hypodermic injections, because of the pain, blood-borne infections^11^, needle-stick and sharp injuries, and biohazardous wastes are likewise not regarded as an ideal delivery system^5,12^. Thus, despite the fact that the barrier properties of the skin reduce the effectiveness of transdermal delivery^13^, it remains the most promising delivery route of biomolecules^14^.

Self-degradable microneedle (DMN) patches, made of arrays of polymeric micron-scale biodegradable needles, were developed as a self-administrable and efficient transdermal delivery platform that penetrates the skin, dissolves, and releases the encapsulated micro- and macro-biopharmaceuticals in a minimally invasive manner without generating any biohazardous wastes^15–18^. DMNs are advantageous compared to orally taken pharmaceuticals as they do not involve the first pass metabolism of the gastrointestinal tract, and are patient friendly in terms of application compared to hypodermic needles as they do not cause pain and possible blood-borne infections^19,20^. Moreover, a vast majority of studies reported that DMNs are more dose-effective than topical and subcutaneous immunization^21,22^.

Although DMNs would overcome most of the limitations associated with conventional transdermal delivery, recent findings suggest that because of the elastic and stiffness properties of the skin^23^, DMNs require secondary supporting structures, applicator systems and, enhancers to achieve an accurate delivery^24–28^. Utilizing a secondary structure such as arrowhead DMNs or pillar-based DMNs^26,29^, however, may increase the chance of tissue damage upon application. Importantly, as the majority of the material is concentrated in the lower half of the DMNs, an incomplete insertion of the array would significantly reduce its delivery effectiveness^15^. On the other hand, in dynamic tissues, any movement including stretching or bending would easily distend DMN from the skin, affecting its delivery efficiency^30,31^. Therefore, an optimized DMN platform that is both simple to fabricate and would effectively deliver the encapsulated compounds is required to overcome the obstacles of the current transdermal delivery systems.

Herein, by focusing on the importance of precise transdermal drug delivery, we introduce tissue interlocking DMN (TI-DMN), which is inspired by the shape of candle flame with a narrow neck, wide body, and sharp tip. Through a series of *in vitro* and *in vivo* evaluations, we systematically demonstrate that TI-DMN accurately delivers the encapsulated drugs to the body, strongly interlocks within the dynamic tissues, and significantly improves the efficiency of delivered compounds compared to conventional DMNs.

## Results

### Fabrication of TI-DMNs

**Figure 1:**
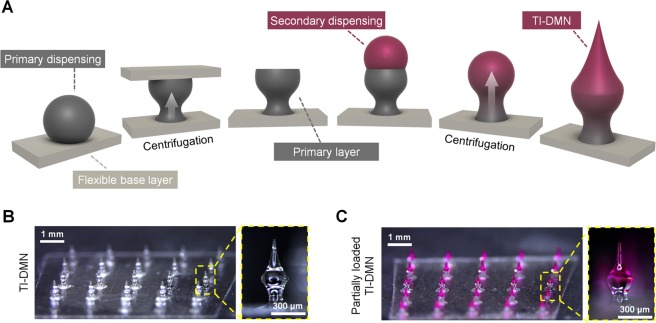
Fabrication of TI-DMNs. (**A**) Schematic representation of TI-DMN fabrication. A polymer droplet is dispensed and centrifuged to form a wineglass-shaped primary layer. An additional droplet is then dispensed over the primary layer followed by a secondary centrifugation to fabricate TI-DMN. Centrifugation force is indicated by white arrow. (**B**) Microscope images of 5 × 5 TI-DMN array. (**C**) Partially loaded TI-DMN array. Scale bars in (**B**) and (**C**) are 1 mm (left panels) and 300 µm (right panels), respectively.

The fabrication process consists of two main steps: the creation of a primary wine glass-shaped layer and a sharp tip secondary layer. Briefly, the viscous hyaluronic acid (HA) polymer is dispensed over a thin solidified layer of HA and set vertically inside a customized centrifuge. Next, a non-sticky Parafilm M coated plate is positioned 200 µm away from the main layer to create a wineglass-shaped primary layer followed by the centrifugation process. Finally, the second HA polymer drop is dispensed on top of the primary layer followed by additional centrifugation, resulting in a TI-DMN with a narrow neck, wide body, and sharp tip **(**Fig. 1A**)**.

We fabricated TI-DMNs with a total height of 600 ± 25 µm in 5 × 5 arrays with the neck, body, and tip diameters at 120 ± 13 µm, 250 ± 28 µm, and 10 ± 6 µm, respectively (Fig. 1B). In addition, conventional DMNs were fabricated with a height of 600 ± 38 µm and tip diameter of 24 ± 7 µm. The encapsulation volume and height of TI-DMNs, based on their target application, can be optimized by adjusting the dispensing volume of polymer drops during the fabrication process. Rhodamine B was utilized as a drug surrogate to evaluate the efficiency of TI-DMNs in delivering encapsulated materials compared with conventional DMNs. The drug surrogate could be loaded within the entire body of TI-DMNs, or partially loaded within the body and tip of TI-DMNs (Fig. 1C).

### Mechanical strength analysis

**Figure 2:**
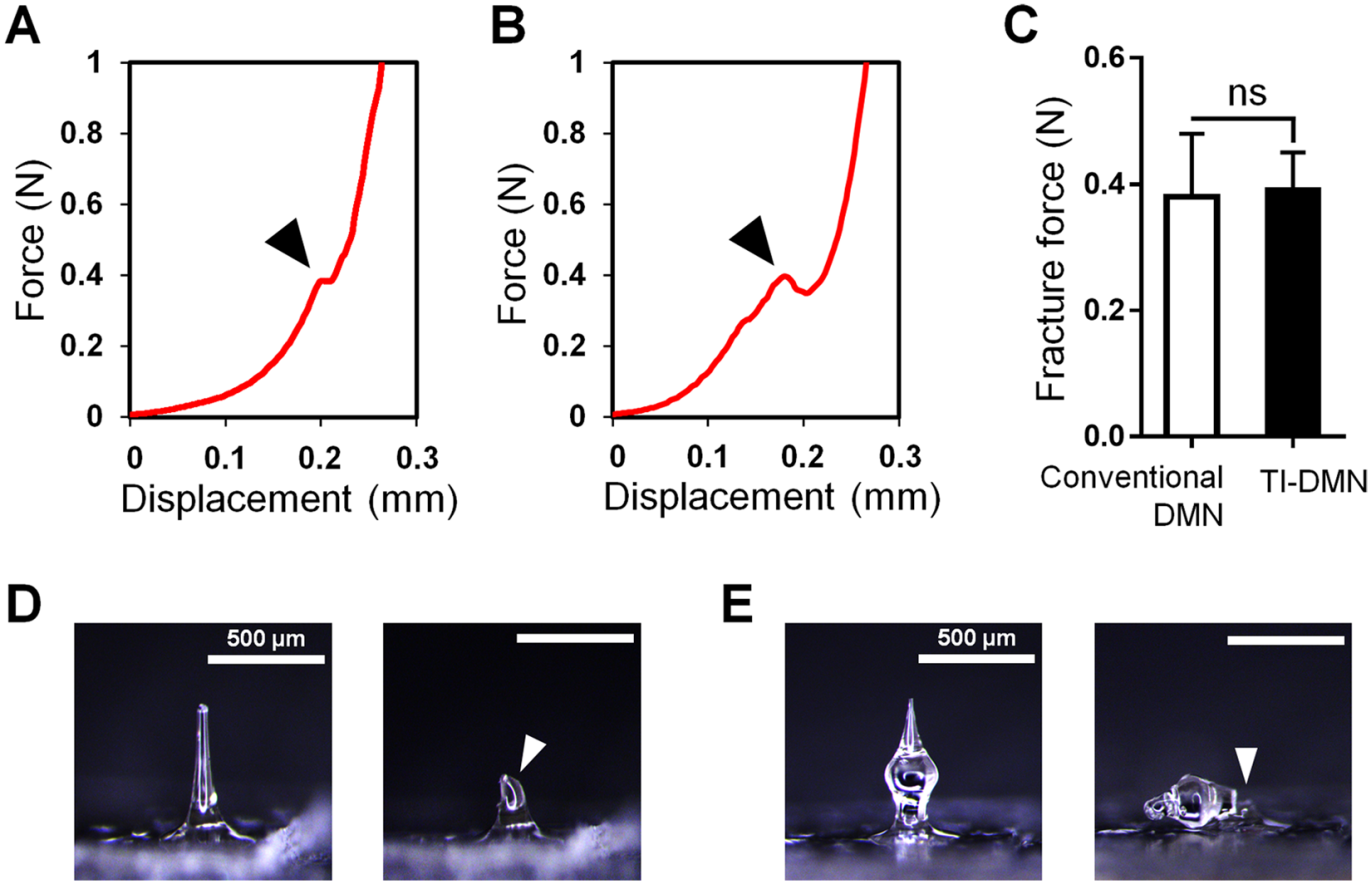
Fracture force evaluation of TI-DMNs compared with conventional DMNs. (**A**) Fracture occurred upon applying 0.38 ± 0.10 N in conventional DMNs and (**B**) 0.39 ± 0.06 N in TI-DMNs. (**C**) There was no significant difference in fracture force of conventional DMNs compared with TI-DMNs. (**D**) Microscope images showed a breakage in the middle of conventional DMNs. (**E**) In TI-DMNs, the breakage occurred at the neck region. Arrows indicate the breakage site. Scale bars in (**D**) and (**E**) are 500 µm.

The minimum force required for a single DMN to penetrate the skin is 0.058 N and any force above that accounts for the ability of DMNs in penetrating the skin^32,33^. Human skin, however, based on the gender, race, and various other factors, poses a unique thickness and elasticity^15,34^. Therefore, to evaluate the penetration capability of TI-DMNs, we first measured the force at which the breakage of a single TI-DMN occurred and compared it with a conventional DMN fabricated with the same volume of materials and height (Fig. 2A,B). Results indicated that the force required to break a single conventional DMN and TI-DMN were 0.38 ± 0.10 N and 0.39 ± 0.06 N, respectively without any significant differences (Fig. 2C). We further evaluated the shape of DMNs prior- and post-breakage and found conventional DMNs broke in the middle whereas TI-DMNs broke in the neck region (Fig. 2D,E). The breakage in the neck region of TI-DMN accounts for its similar fracture force with conventional DMNs Overall, these results implied that the mechanical strength of TI-DMN is sufficient to successfully penetrate the skin without breakage.

### Skin penetration and attachment force evaluation

**Figure 3:**
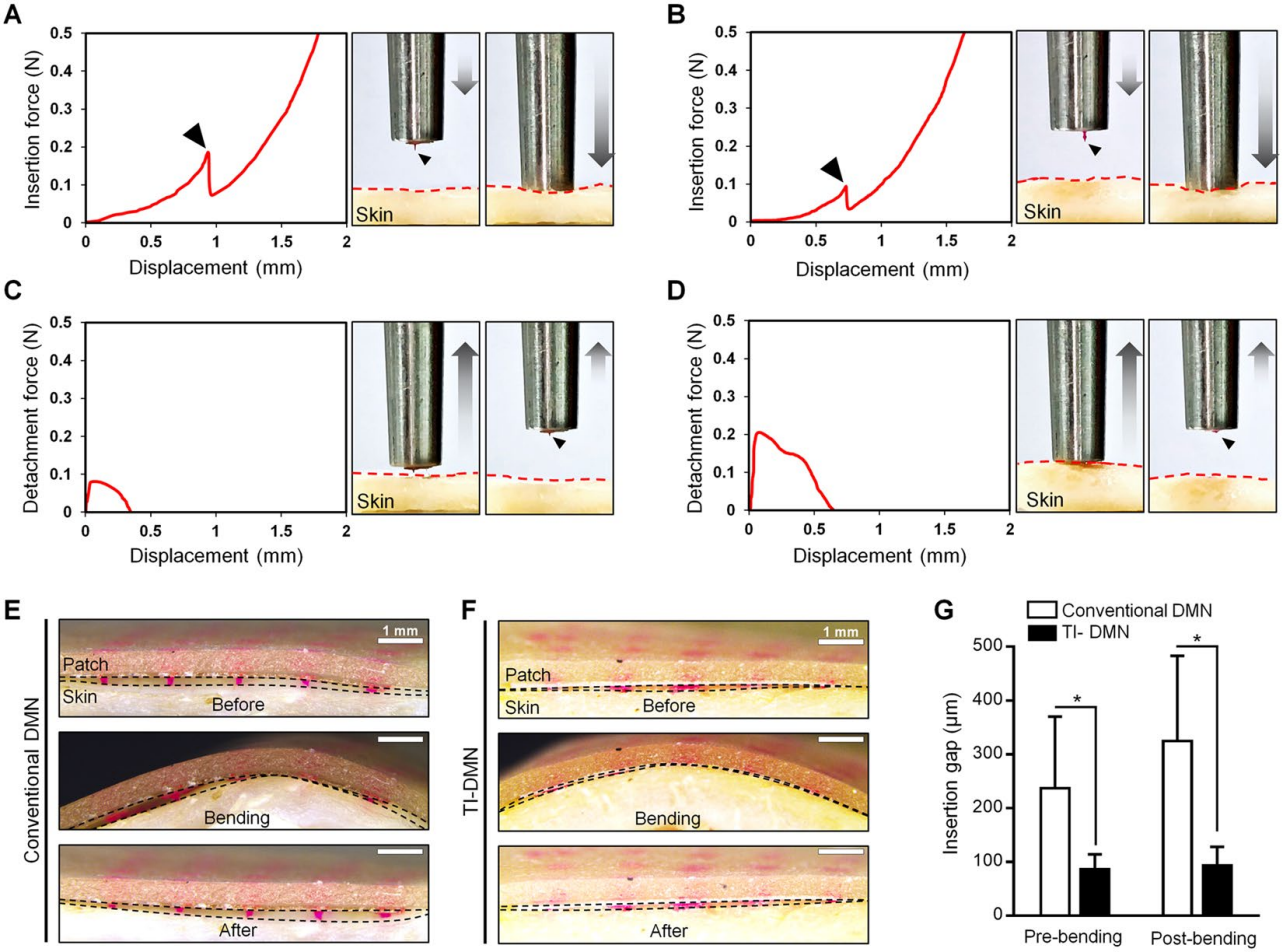
Tissue penetration and attachment analysis of TI-DMNs. (**A**) Pig cadaver skin was pierced at 0.19 ± 0.06 N in conventional DMNs, (**B**) and 0.1 ± 0.07 N in TI-DMNs. The skin detachment force required (**C**) for conventional DMNs was 0.08 ± 0.05 N at a short displacement distance of 0.35 ± 0.1 mm, whereas (**D**) for TI-DMN, it was 0.2 ± 0.11 N at 0.66 ± 0.13 mm. (**E**) Comparison of dynamic tissue interlocking ability of conventional DMNs compared with (**F**) TI-DMNs using pig cadaver skin. (**G**) The insertion gap between DMNs and skin was significantly larger in conventional DMNs at 237 ± 133 µm compared with TI-DMNs at 86 ± 28 µm. After 10 bending cycles at 120°, while the gap was maintained in TI-DMN patches, it was further increased in conventional DMNs. Data in (**G**) are the mean ± s.e.m (n = 5).

Skin penetration and attachment quality of DMNs is among the key factors determining delivery accuracy and efficiency of encapsulated biopharmaceuticals^15,20,24^. Thus, to achieve a precise delivery, DMNs must be completely embedded and interlocked within the tissues throughout the dissolution process. To assess the skin penetration characteristics of TI-DMNs, we measured the force required to pierce a pig cadaver skin (Fig. 3A,B). The minimum force required to penetrate the skin was 0.1 ± 0.07 N and 0.19 ± 0.06 N for a single TI-DMN and conventional DMN, respectively, with no statistically significant difference (Supplementary Fig. 1). As previously reported, a lower skin insertion force with shorter displacement suggests a smoother skin penetration^35^. However, to evaluate the skin insertion characteristic of TI-DMNs in detail, a series of quantitative measurements as well as high-magnification microscopy record of insertion process are remained to be addressed in the future research. Overall, based on above findings, we conclude that similar with conventional DMNs, TI-DMNs penetrate the skin in a minimally invasive manner^22,26^.

Our hypothesis was that the combination of narrow neck and wide body of TI-DMNs function as a physical interlocking tool in the dynamic skin. Thus, to confirm the tissue interlocking feature of DMNs, we measured the force required to detach a TI-DMN from the skin and compared it with conventional DMNs (Fig. 3C,D). We found that at 5 min post application, TI-DMNs required a minimum detachment force of 0.2 ± 0.11 N to separate from the skin, whereas to separate a conventional DMN, a lower force of 0.08 ± 0.05 N was required (Supplementary Fig. 1). Interestingly, conventional DMNs were immediately detached from the skin with only 0.35 ± 0.1 mm displacement, whereas the minimum displacement for TI-DMN was 0.66 ± 0.13 mm. The longer displacement accounts for the stronger interlocking within the tissues, suggesting TI-DMNs would interlock within the skin more strongly than conventional DMNs.

The likelihood of incomplete insertion increases in dynamic tissues because of the stretching or bending motions of the skin^36,37^. Therefore, to evaluate attachment efficiency of TI-DMNs in dynamic tissues, we applied 5 × 5 arrays of TI-DMN on pig cadaver skin and compared the gap created upon 10 cycles of bending motion at 120° angle with conventional DMNs. Results indicated that conventional DMNs were not completely embedded into the skin and a gap of 237 ± 133 µm was present between the arrays and the tissue upon application. In the TI-DMN patches, a significantly smaller gap of 86 ± 28 µm was observed, indicating a remarkably improved insertion quality (Fig. 3E,F). Although both DMNs were inserted to the same depth of the skin, the gap was significantly smaller in TI-DMNs because of their interlocking geometry. Under the cyclic bending motion, we found that while the gap in conventional DMN group was increased to 325 ± 158 µm, TI-DMNs remained strongly interlocked within the skin without any significant changes (Fig. 3G). These findings confirmed that the narrow neck to wide body morphology of TI-DMNs creates an interlocking functionality that prevents them from skin detachment regardless of skin bending or stretching.

### Volume distribution and diffusion evaluation

**Figure 4:**
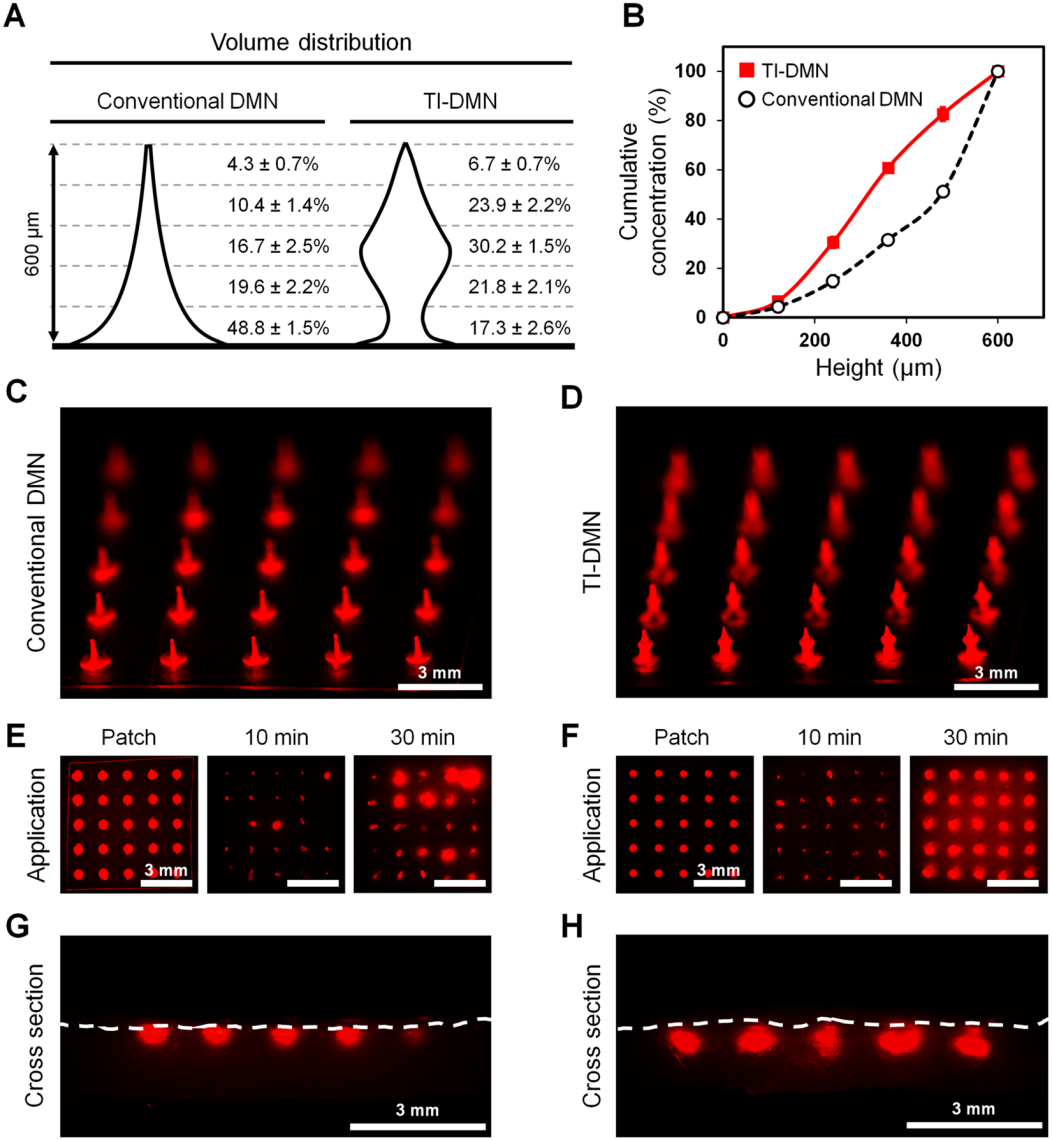
Volume distribution and diffusion pattern of TI-DMN. (**A**) Volume distribution comparison of conventional DMNs and TI-DMNs. (**B**) Cumulative volume distribution confirmed the main volume of drug surrogate was concentrated in the mid-portion of TI-DMNs. (**C**) A 5 × 5 array of conventional DMNs compared with (**D**) TI-DMNs. (**E**) At 30 min post application, while diffusion pattern of conventional DMNs varied in each spot, (**F**) it was highly uniform in TI-DMNs. (**G**) Cross section of skin showed drug surrogate was localized at the outermost layer of skin in conventional DMNs, (**H**) whereas it was diffused beneath the skin in TI-DMNs. Data in (**A**), and (**B**) are the mean ± s.e.m (n = 5). Scale bars are 3 mm for (**C**–**H**).

As the probability of incomplete insertion is notably high in DMNs, distribution of encapsulated compounds would directly affect the delivery efficiency. To quantitatively assess the drug volume distribution, we utilized an online software program to replicate the shape of DMNs and measure their volume accordingly. Both conventional DMNs and TI-DMNs were fabricated at the same height using the same volume of polymer mixtures (Fig. 4A). Volume analysis data suggested that in conventional DMNs 48.8 ± 1.5% of drug surrogate was encapsulated up to 120 µm from the base, whereas this was only 17.3 ± 2.6% in TI-DMNs. These results suggest that even in the case of an incomplete insertion in which 120 µm of DMNs are not inserted into the skin, TI-DMN would be capable of delivering a significantly higher concentration of encapsulated biopharmaceuticals compared to conventional DMNs. Next, based on the volume distribution data, we investigated the cumulative concentration of DMNs (Fig. 4B, Supplementary Fig. 2). At 240 µm from the tip, the cumulative concentration of TI-DMN was approximately double than that of the conventional DMNs at 30.6 ± 2% and 14.7 ± 2.1%, respectively. This significant difference was maintained up to 480 µm from tip at 82.65 ± 2.6% in TI-DMNs and 51.1 ± 1.5% in conventional DMNs. Based on above findings, we confirmed a high volume of drug surrogate is concentrated in the lower body of conventional DMNs, leading to a drastically reduced delivery volume in case of incomplete insertion, whereas in TI-DMNs, the major volume of materials are localized in the mid-body, resulting in a highly accurate delivery (Supplementary Fig. 3**)**.

To evaluate diffusion pattern of TI-DMNs, we assessed the *in vitro* release intensity profile of TI-DMN over the pig cadaver skin in comparison to that of conventional DMNs (Fig. 4C,D). At 30 min post application, a considerably higher intensity was detected in TI-DMN treated group than in conventional DMNs (Fig. 4E,F). Unlike conventional DMN, we confirmed a highly uniform diffusion pattern in TI-DMN treated skin. These results suggested the enhanced delivery accuracy of TI-DMNs compared with conventional DMNs. To investigate the permeation pattern of TI-DMNs across the skin, the tissues were sectioned at the mid-point of each array. Data indicated that the drug surrogate in conventional DMN-treated skin was dissolved and localized at the outermost layer of the skin at 488 ± 61 µm below the epidermis (Fig. 4G, Supplementary Fig. 4). Additionally, conventional DMNs were not completely inserted into the skin, leading to distinct diffusion of fluorescence intensities in an array. Interestingly, unlike in conventional DMNs where the diffusion was localized at the epidermis, TI-DMNs were primarily diffused beneath the skin beginning from 125 ± 86 µm to 602 ± 47 µm (Fig. 4H, Supplementary Fig. 4). These findings confirmed TI-DMNs are advantageous as they encapsulate the major volume of materials in their mid-body, increasing the delivery efficiency compared with conventional DMNs.

### Transcutaneous permeation analysis

**Figure 5:**
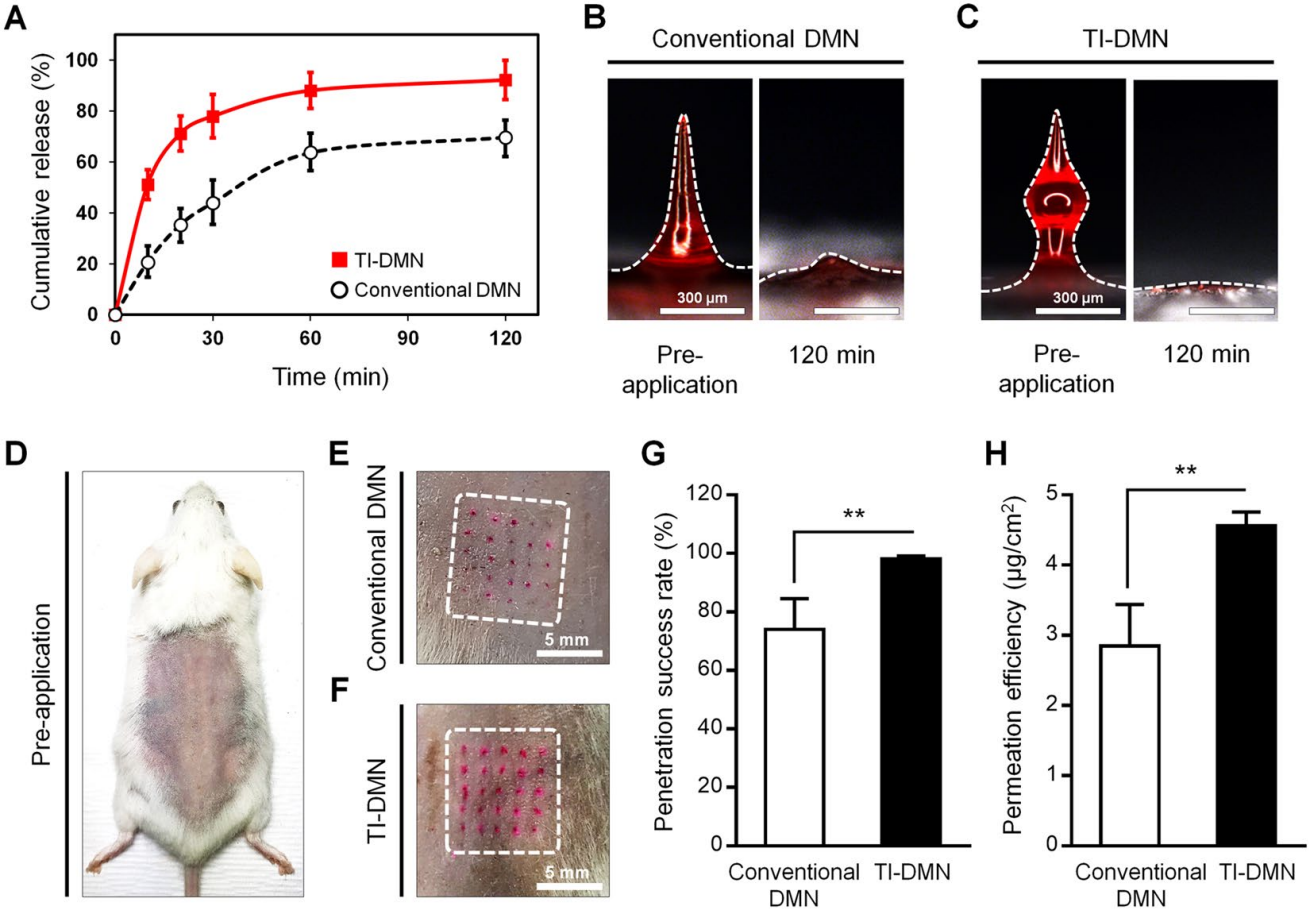
Penetration and permeation analysis of TI-DMNs compared with conventional DMNs. (**A**) Transcutaneous permeation kinetic analysis confirmed that TI-DMN delivered a larger volume of encapsulated drug surrogate at a faster rate compared to conventional DMN. (**B**) A remarkably larger portion of encapsulated drug surrogate remained undissolved over the patch in conventional DMN compared with (**C**) TI-DMNs post application. (**D**) The dorsal skin of mice prior to DMN application. (**E**) While the diffusion spots in conventional DMN-treated skin were barely visible, (**F**) a remarkably higher intensity was detected in TI-DMN-treated mice. (**G**) TI-DMN posed a penetration success rate of 98 ± 1.1% compared to conventional DMNs at 74 ± 5.2%. (H) TI-DMN delivered a notably higher volume of the encapsulated drug surrogate compared to conventional DMNs. Data in (**A**), (**G**), and (**H**) are the mean ± s.e.m (n = 4). Scale bars in (**B**) and (**C**) are 300 µm, and in (**E**) and (**F**) are 5 mm.

To assess transcutaneous permeation kinetics of drug surrogate encapsulated TI-DMNs, we utilized a diffusion cell apparatus, which mimics the biological environment of human skin *in vitro*^38,39^. DMNs were applied on pig cadaver skin placed over the diffusion cell apparatus and measured the amount of delivered drug surrogate up to 120 min. Analyzing the release profile pattern of drug surrogate confirmed that TI-DMN induced a faster drug surrogate accumulation compared to conventional DMN (Fig. 5A). Likewise, a significantly higher volume was delivered by TI-DMN compared to conventional DMN at 92.2 ± 13.3% and 69.7 ± 11.8%, respectively. As shown in Fig. 4A, the fact that most of the drug surrogate is concentrated in the lower body of conventional DMNs, is assumed to be responsible for their poor delivery efficiency. DMNs without drug surrogate were employed as controls and were not detected during the experiment. Further investigation showed that while the base portion of conventional DMN remained undissolved over the patch, TI-DMNs were almost completely dissolved at 120 min (Fig. 5B,C). These findings confirmed that TI-DMN delivers the encapsulated materials more effectively and at a faster rate than conventional DMNs.

To investigate skin penetration characteristics of TI-DMN arrays *in vivo*, we applied them on the dorsal skin of mice for 120 min and compared their insertion success rate and permeation volume to conventional DMN patches (Fig. 5D). Photographs of dorsal skin showed a complete array of spots in the mice treated with TI-DMN patch, whereas in conventional DMNs the spots were barely visible, suggesting conventional DMNs were either not completely inserted inside the skin or not inserted at all (Fig. 5E,F). A considerably higher diffusion intensity was detected in the group treated with TI-DMN than with conventional DMN.

Next, we investigated the insertion success rate of TI-DMNs *in vivo*. Regardless of completeness or quality of insertion, the total number of DMNs in an array that pierced the skin were counted. We found that the penetration success rate of TI-DMNs at 98 ± 1.1% was considerably higher than conventional DMNs at 74 ± 5.2% (Fig. 5G). These data implied that compared to TI-DMNs in which the whole array was inserted into the skin, 24 ± 5.4% of conventional DMNs were remained untouched throughout the application process.

Finally, to assess the permeation efficiency of TI-DMNs *in vivo*, we measured the volume of drug surrogate that permeated into the skin for 120 min. TI-DMN treated group delivered a significantly higher volume of drug surrogate compared to conventional DMNs at 4.56 ± 0.19 µg/cm^2^ and 2.85 ± 0.29 µg/cm^2^, respectively (Fig. 5H). The improved permeation efficiency of TI-DMN was mainly due to combination of the interlocking geometry and encapsulation of the major volume of drug surrogate in its mid-body. DMNs without the drug surrogate showed no permeation throughout the experiment. Therefore, we conclude that TI-DMNs were capable of delivering the encapsulated compounds with a high efficiency beneath the skin. This plays a crucial role for those pharmaceuticals that their precise delivery is important to achieve an aimed biological feedback from the body.

## Discussion

The TI-DMN assessed in this study is a platform for accurate, effective, and efficient transdermal delivery of biomolecules, developed to mitigate the limitations associated with conventional DMNs. Our findings confirmed that the physical properties of skin prevents conventional DMNs from complete insertion, leading to inefficient and inaccurate delivery of encapsulated compounds. Therefore, various types of applicators and chemical formulations were incorporated to overcome the obstacles associated with incomplete skin insertion of DMNs. Likewise, in dynamic tissues, any sort of movement including bending or stretching could easily distend and detach DMNs from the skin. TI-DMNs, however, by combining a narrow neck and a wide body shape, were shown to completely penetrate the skin, accurately deliver the encapsulated biomolecules, and prevent the possible detachment from the skin during the application process.

The fabrication process of TI-DMNs using centrifugal lithography takes up to 10 min and up to 4 arrays of 8 × 8 TI-DMNs can be fabricated during each centrifugation cycle. While HA was employed for fabrication of both primary and secondary layers of TI-DMN in this study, other widely employed polymers including carboxymethlycellulose, alginate, dextrin, Poly(vinyl alcohol) can be utilized as dissolvable backbone polymer of TI-DMNs.

TI-DMN is advantageous as the whole array penetrates the skin uniformly and delivers biomaterials with higher accuracy compared to conventional DMN. This not only accelerates the biological feedback response of the body, but it also improves the delivery efficiency of encapsulated materials.

DMNs are subjected to the “bed of nails” effect^40^, in which the force is evenly distributed across the whole array, resulting in incomplete skin insertion and reduced delivery efficiency of DMNs^41^. Therefore, in addition to the shape of DMNs, the distance between each DMN and the total number of DMNs in an array may also play an important role in delivery efficiency of encapsulated biomolecules^41,42^, which remains to be addressed in further research.

Throughout the study, pig cadaver skin has been used as an *in vitro* model. Comparing to the human skin, pig cadaver skin exhibits similar composition and stiffness properties. However, we should consider the fact that the skin hydration rate and the difference in skin stiffness would highly affect the insertion and interlocking ability of TI-DMNs. Moreover, as positioning pig cadaver skin over the Franz cell diffusion system does not completely mimic the actual environment of body, the dissolution rate and permeation pattern of DMN is expected to be different in clinical application.

It is important to consider that while we utilized rhodamine B as the drug surrogate for the proof-of-concept in this study, TI-DMNs are capable of encapsulating various kind of micro- and macro-biomolecules including vaccines, proteins, and peptides. Thus, considering the advancements and advantages of TI-DMNs compared to conventional DMNs, this novel system would be an ideal platform for transdermal drug delivery in the future.

## Conclusions

In conclusion, we have demonstrated a TI-DMN transdermal delivery platform that smoothly penetrates the skin, interlocks within tissues, and effectively delivers the encapsulated compounds. TI-DMNs are composed of a narrow neck, wide body, and sharp tip that improves their skin insertion capability and reduces the chances of detachment from the skin prior to dissolution. Based on a series of *in vitro* and *in vivo* evaluations, we confirmed TI-DMN exhibits a significantly higher delivery accuracy and efficiency of encapsulated materials compared to conventional DMNs.

## Methods

### Fabrication process of TI-DMN arrays

Polymer solutions were prepared by mixing 60% HA (32 kDa, Soliance, Pomacle, France) powder in distilled water at 32 °C. Rhodamine B (0.1% w/w, 479 Da, Sigma Aldrich, MO, USA) was employed as the drug surrogate. To fabricate DMNs, we employed centrifugal lithography^43^, a recently introduced economical and simple fabrication method. In brief, to fabricate TI-DMNs, polymer droplets were dispensed in 5 × 5 arrays (SHOT mini 100-s, Musashi, Tokyo, Japan) over a flexible plate, positioned 200 µm away from a non-sticky plate and a centrifugal force of 300 *g* was applied to elongate and form a wineglass-shaped polymer structure. Next, the non-sticky plate was removed, and secondary polymer droplet was dispensed on top of the primary wine glass-shaped layer. Finally, a centrifugal force of 400 *g* was applied for 1 min to form TI-DMN arrays with a height of 600 ± 25 µm. The conventional DMNs were fabricated at the same height and polymer volume using centrifugal lithography by a centrifugal force of 500 *g* for 1 min.

### Measurement of fracture and skin penetration forces

Mechanical fracture force of DMNs was measured using Z0.5TN (Zwick/Roell, Ulm, Germany) at a speed of 1 mm/min. DMNs were positioned vertically against a sensor probe, which pressed DMNs and measured their fracture force with regard to displacement. Skin insertion force was measured by attaching a single DMN on the sensor probe and pressing it at a speed of 1 mm/min toward a pig cadaver skin (surface area: 2.5 cm^2^, thickness: 1 mm; CRONEX, Seoul, South Korea). Likewise, the detachment force was measured by removing DMN from the skin at 5 min post application.

### Dynamic tissue attachment analysis

DMNs fabricated in 5 × 5 arrays were attached on a sticky patch and applied onto a pig cadaver skin with the surface area of 2.5 cm^2^ and thickness of 1 mm (n = 5/group). The initial gap between skin and the patch was measured by calculating the average of 5 fixed points per sample using a M165 FC bright-field optical microscope (Leica, Wetzlar, Germany). Next, the skin was bent and stretched 10 times at 120° to mimic a dynamic tissue *in vitro*. The bending process was performed using a custom-made tool to ensure the skin does not bend more or less than the dedicated angle. Accordingly, the final gap between the patch and skin was measured and compared to the initial gap.

### Cutaneous permeation kinetics

Diffusion of DMNs were analyzed using a Franz cell diffusion chamber (SES GmbH) equipped with a stirrer set at 250 rpm and temperature control system set at 32 ± 1 °C to mimic the blood circulation in the body *in vitro*. The diffusion of rhodamine B loaded DMNs were separately visualized over the epidermis of pig cadaver skin at 10 and 30 min. At 30 min post application, the tissues were sectioned to visualize the permeation pattern and localization of diffusion within the skin.

Next, the skin permeation kinetics of drug surrogate encapsulated DMNs was evaluated up to 120 min. At 120 min, the skin samples were soaked into the chamber and stirred for an additional 2 h. The total volume of delivered drug surrogate at 4 h was set as maximum and the permeated volume of each sample was calculated relatively. DMNs without the drug surrogate were used as controls. The intensity of permeated drug surrogate in each sample was measured using a multimode plate reader (VICTOR™ X, PerkinElmer).

### DMN volume distribution calculation

Both conventional DMN and TI-DMNs were fabricated at the same height with the exact same volume of polymer. As the shape of each DMN varied slightly, we selected 5 samples per group and calculated their areas. To calculate the drug distribution, we employed a software (SketchAndCalc™), loaded the microscope images of DMNs, and synchronized the scale bars with the software for an accurate simulation. The area of DMNs was then measured from tip to base by dividing each DMN into 5 sections of 120 µm.

### *In vivo* insertion and permeation analysis

Male BALB/c mice (6 weeks old) were purchased from Orient Bio (Gyeonggi-do, South Korea) and given one week to adapt to the environment. The dorsal skin of mice was shaved using an electric razor and the drug surrogate encapsulated DMNs were applied for up to 120 min (n = 4/group). To measure the permeation efficiency, the experimented mice skin was cut, embedded in Franz cell diffusion chamber, and stirred at 32 ± 1 °C for 2 h. The intensity of the samples was measured using the multimode plate reader.

To measure the penetration success rate, we counted the number of detectable pierced spots at 120 min post application using the microscope. The quality and depth of insertion were disregarded. All procedures were approved and performed in accordance with the guidelines and regulations of the experimentation ethics by Yonsei Laboratory Animal Research Centre (YLARC).

### Statistical analysis

Means were compared using Student’s *t*-test or one-way analysis of variance (ANOVA) using GraphPad Prism 6 software. P-values of <0.05 were considered significant.

## Supplementary information

Supplementary Information

## Data Availability

All relevant data are available within the manuscript and supplementary information.

## References

[1] Moga KA (2013). Rapidly-dissolvable microneedle patches via a highly scalable and reproducible soft lithography approach. Adv Mater.

[2] Lahiji SF, Dangol M, Jung H (2015). A patchless dissolving microneedle delivery system enabling rapid and efficient transdermal drug delivery. Sci Rep.

[3] Kim YC, Ludovice PJ, Prausnitz MR (2010). Transdermal delivery enhanced by antimicrobial peptides. J Biomed Nanotechnol.

[4] Wertz PW (2000). Lipids and barrier function of the skin. Acta Derm Venereol Suppl (Stockh).

[5] Sullivan SP, Murthy N, Prausnitz MR (2008). Minimally invasive protein delivery with rapidly dissolving polymer microneedles. Adv Mater.

[6] Kim YC, Prausnitz MR (2011). Enabling skin vaccination using new delivery technologies. Drug Deliv Transl Res.

[7] Deng Y (2016). Transdermal Delivery of siRNA through Microneedle Array. Sci Rep.

[8] Bos JD, Meinardi MM (2000). The 500 Dalton rule for the skin penetration of chemical compounds and drugs. Exp Dermatol.

[9] Lee JW, Park JH, Prausnitz MR (2008). Dissolving microneedles for transdermal drug delivery. Biomaterials.

[10] Pond SM, Tozer TN (1984). First-pass elimination. Basic concepts and clinical consequences. Clin Pharmacokinet.

[11] Simonsen L, Kane A, Lloyd J, Zaffran M, Kane M (1999). Unsafe injections in the developing world and transmission of bloodborne pathogens: a review. Bull World Health Organ.

[12] Prausnitz MR, Mitragotri S, Langer R (2004). Current status and future potential of transdermal drug delivery. Nat Rev Drug Discov.

[13] Chen Y (2006). Transdermal protein delivery by a coadministered peptide identified via phage display. Nat Biotechnol.

[14] Mitragotri S, Burke PA, Langer R (2014). Overcoming the challenges in administering biopharmaceuticals: formulation and delivery strategies. Nat Rev Drug Discov.

[15] Lee JW, Choi SO, Felner EI, Prausnitz MR (2011). Dissolving microneedle patch for transdermal delivery of human growth hormone. Small.

[16] Zhu Z (2014). Rapidly dissolvable microneedle patches for transdermal delivery of exenatide. Pharm Res.

[17] Kaushik S (2001). Lack of pain associated with microfabricated microneedles. Anesth Analg.

[18] Pan J (2018). Intradermal delivery of STAT3 siRNA to treat melanoma via dissolving microneedles. Sci Rep.

[19] Lee K, Jung H (2012). Drawing lithography for microneedles: A review of fundamentals and biomedical applications. Biomaterials.

[20] Fakhraei Lahiji S (2018). Transcutaneous implantation of valproic acid-encapsulated dissolving microneedles induces hair regrowth. Biomaterials.

[21] Van Damme P (2009). Safety and efficacy of a novel microneedle device for dose sparing intradermal influenza vaccination in healthy adults. Vaccine.

[22] van der Maaden K, Jiskoot W, Bouwstra J (2012). Microneedle technologies for (trans)dermal drug and vaccine delivery. J Control Release.

[23] Donnelly RF (2010). Optical coherence tomography is a valuable tool in the study of the effects of microneedle geometry on skin penetration characteristics and in-skin dissolution. J Control Release.

[24] Chen MC, Huang SF, Lai KY, Ling MH (2013). Fully embeddable chitosan microneedles as a sustained release depot for intradermal vaccination. Biomaterials.

[25] Ita K (2017). Dissolving microneedles for transdermal drug delivery: Advances and challenges. Biomed Pharmacother.

[26] Chu LY, Prausnitz MR (2011). Separable arrowhead microneedles. J Control Release.

[27] Karande P, Jain A, Mitragotri S (2004). Discovery of transdermal penetration enhancers by high-throughput screening. Nat Biotechnol.

[28] Williams AC, Barry BW (2004). Penetration enhancers. Adv Drug Deliv Rev.

[29] Chen J (2018). Fabrication of Tip-Dissolving Microneedles for Transdermal Drug Delivery of Meloxicam. AAPS PharmSciTech.

[30] Yang SY (2013). A bio-inspired swellable microneedle adhesive for mechanical interlocking with tissue. Nat Commun.

[31] Bal SM (2010). Influence of microneedle shape on the transport of a fluorescent dye into human skin *in vivo*. J Control Release.

[32] Davis SP, Landis BJ, Adams ZH, Allen MG, Prausnitz MR (2004). Insertion of microneedles into skin: measurement and prediction of insertion force and needle fracture force. J Biomech.

[33] Kim JD, Kim M, Yang H, Lee K, Jung H (2013). Droplet-born air blowing: novel dissolving microneedle fabrication. J Control Release.

[34] Andrianov AK, Marin A, DeCollibus DP (2011). Microneedles with intrinsic immunoadjuvant properties: microfabrication, protein stability, and modulated release. Pharm Res.

[35] Romgens AM, Bader DL, Bouwstra JA, Baaijens FPT, Oomens CWJ (2014). Monitoring the penetration process of single microneedles with varying tip diameters. J Mech Behav Biomed Mater.

[36] Enfield J (2010). *In-vivo* dynamic characterization of microneedle skin penetration using optical coherence tomography. J Biomed Opt.

[37] Moronkeji K, Todd S, Dawidowska I, Barrett SD, Akhtar R (2017). The role of subcutaneous tissue stiffness on microneedle performance in a representative *in vitro* model of skin. J Control Release.

[38] Desmedt B (2015). *In vitro* Dermal Absorption: Sample Application and Seal Quality in a Franz Diffusion Cell System. Skin Pharmacol Physiol.

[39] Seo JE, Kim S, Kim BH (2017). *In vitro* skin absorption tests of three types of parabens using a Franz diffusion cell. J Expo Sci Environ Epidemiol.

[40] Verbaan FJ (2008). Improved piercing of microneedle arrays in dermatomed human skin by an impact insertion method. J Control Release.

[41] Olatunji O, Das DB, Garland MJ, Belaid L, Donnelly RF (2013). Influence of array interspacing on the force required for successful microneedle skin penetration: theoretical and practical approaches. J Pharm Sci.

[42] Chen Z (2018). Additive Manufacturing of Honeybee-Inspired Microneedle for Easy Skin Insertion and Difficult Removal. ACS Appl Mater Interfaces.

[43] Yang Huisuk, Kim Suyong, Kang Geonwoo, Lahiji Shayan F., Jang Mingyu, Kim Young Mi, Kim Jae-Myung, Cho Sang-Nae, Jung Hyungil (2017). Centrifugal Lithography: Self-Shaping of Polymer Microstructures Encapsulating Biopharmaceutics by Centrifuging Polymer Drops. Advanced Healthcare Materials.

