# Supplementary material for: Tissue Interlocking Dissolving Microneedles for Accurate and Efficient Transdermal Delivery of Biomolecules
Source: Sci Rep. 2019 May 27;9:7886. doi: 10.1038/s41598-019-44418-6 (PMC6536679; doi:10.1038/s41598-019-44418-6)
Supplement: Supplementary file 1 — Supplementary Information [file 41598_2019_44418_MOESM1_ESM.pdf]

Tissue Interlocking Dissolving Microneedles for Accurate and Efficient Transdermal Delivery of Biomolecules

Shayan Fakhraei Lahiji[1], Youseong Kim[1], Geonwoo Kang[1,2], Suyong Kim[1], Seunghee Lee[1], Hyungil Jung[1,2,*]

[1] Department of Biotechnology, Building 123, Yonsei University, 50 Yonsei-ro, Seodaemun-gu, Seoul, 03722, Republic of Korea

[2] Juvic Inc., 272 Digital-ro, Guro-gu, Seoul 08389, Republic of Korea

*Corresponding author: hijung@yonsei.ac.kr, Phone: 82-2-2123-7418, Fax: 82-2-362-7265

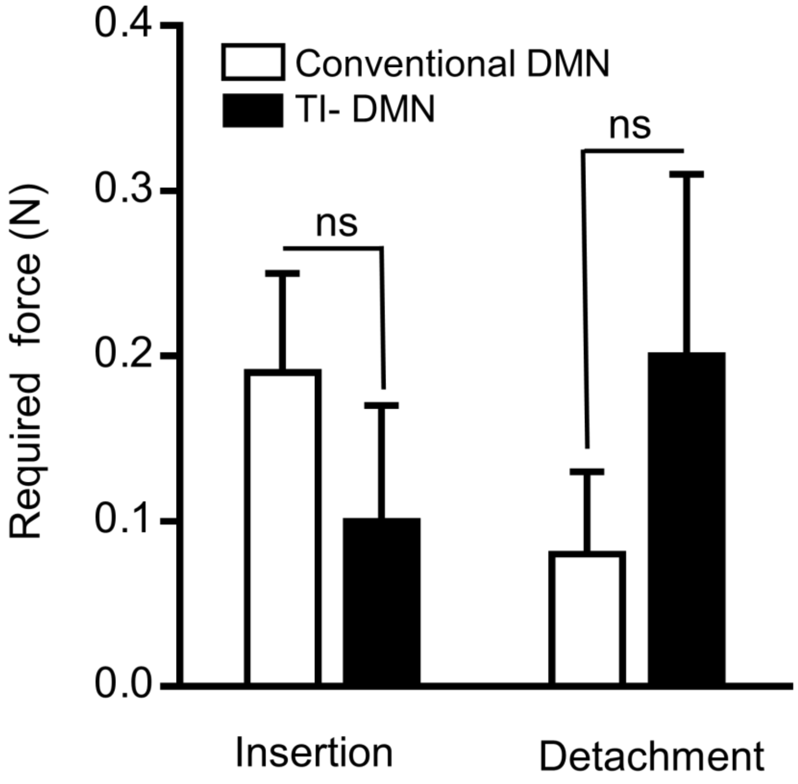

Supplementary Figure 1. Skin insertion and detachment of DMNs. Results indicated less force required in skin penetration using TI-DMNs compared with conventional DMNs and stronger interlocking within the skin in TI-DMNs. Due to physical properties of pig cadaver skin, achieving a significant result was not possible. Data are the mean ± s.e.m (n = 5).

Height / percentage	Conventional DMN		TI-DMN	
120 μm / 20%	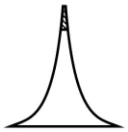	4.3 ± 0.7%	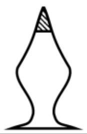	6.7 ± 0.7%
240 μm / 40%	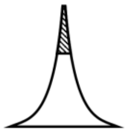	14.7 ± 2.1%	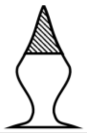	30.6 ± 2%
360 μm / 60%	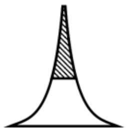	31.5 ± 1.6%	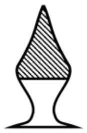	60.8 ± 0.4%
480 μm / 80%	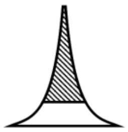	51.1 ± 1.5%	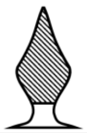	82.6 ± 2.6%
600 μm / 100%	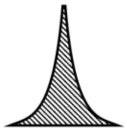	100%	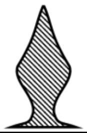	100%

Supplementary Figure 2. Encapsulation capacity analysis based on height of DMNs. Up to 480 μm, conventional DMNs encapsulated 51.1 ± 1.5% of materials, whereas TI-DMN was capable of encapsulating a significantly higher volume of 82.6 ± 2.6%. TI-DMNs, therefore, are capable of delivering higher volume of encapsulated materials compared with conventional DMNs, in case of incomplete insertion.

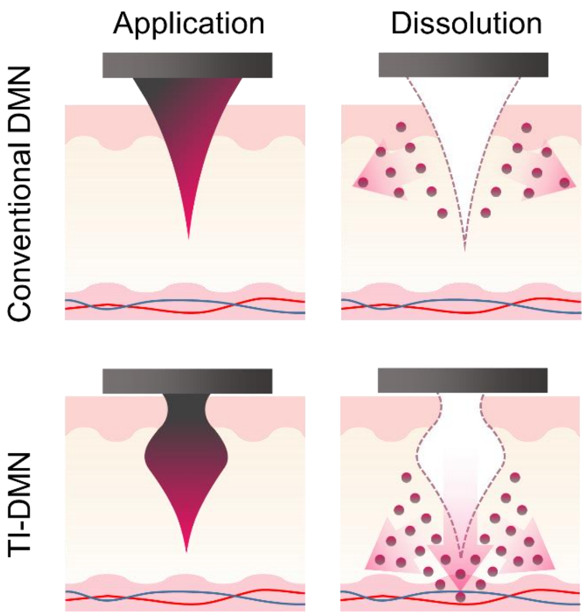

Supplementary Figure 3. An illustration comparing the dissolution efficiency of TI-DMNs and conventional DMNs. The major volume of encapsulated drug surrogate is concentrated in the mid-body of TI-DMNs. Moreover, while conventional DMNs are not completely inserted into the skin, TI-DMNs are fully embedded within the tissues, leading to a significantly efficient delivery of encapsulated biopharmaceuticals.

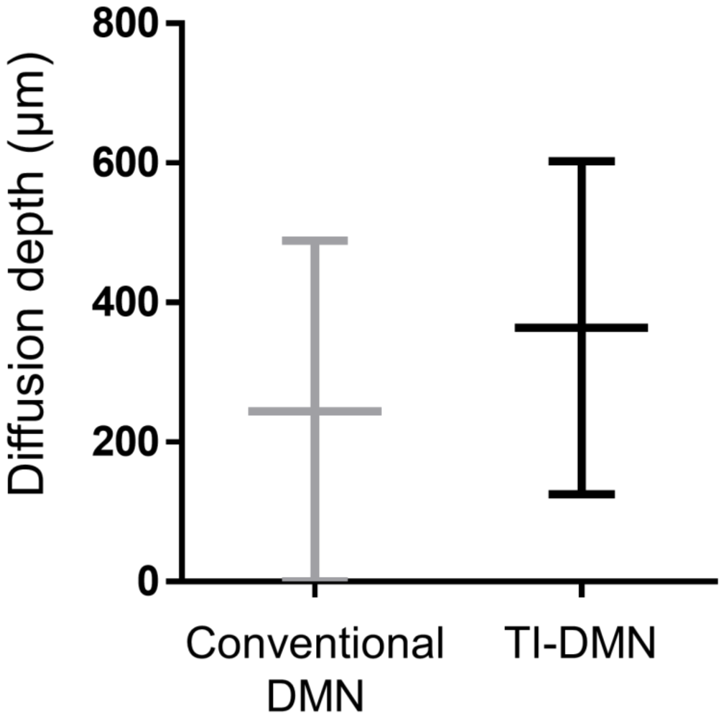

Supplementary Figure 4. Diffusion depth of drug surrogate within the skin. Drug surrogate was diffused at the outermost layer of skin up to 488 ± 61 µm below the epidermis in conventional DMNs, whereas the diffusion started from 125 ± 86 µm to 602 ± 47 µm below the skin in TI-DMN treated group. Data are plotted as min to max (n = 5).
